# Supply and demand barriers to PHC maternal care services uptake: qualitative and behavioural insights from Gombe State, Nigeria

**DOI:** 10.1186/s12884-025-08071-4

**Published:** 2025-09-24

**Authors:** Mohammed M. Alhaji, Laila Umar, Maryam Anike Yusuf, Robert Nyaga, Jaspreet Singh, Arizechukwu Okafor, Francis Meyo, Zarah Haruna Shayau, Zainab Ibrahim Isah, Maryam Abubakar, Aisha Isa Umar, Yakubu Ozohu-Suleiman

**Affiliations:** 1 Busara Centre for Behavioural Economics (Busara), Abuja, Nigeria; 2 National Primary Health Care Development Agency (NPHCDA), Abuja, Nigeria; 3 Busara Centre for Behavioural Economics (Busara), Nairobi, Kenya; 4 Busara Centre for Behavioural Economics (Busara), New Delhi, India; 5 Gombe State Primary Health Care Development Agency (SPHCDA), Gombe, Nigeria

**Keywords:** Behaviour, Health-seeking, Maternal care, Antenatal care, Family planning, Cognitive biases, Barriers, Supply-side, Demand-side, Social ecological model (SEM)

## Abstract

**Background:**

Several factors influence maternal morbidity and mortality in northeast Nigeria, and maternal care-seeking behaviour at primary healthcare facilities is a critical factor. This study, rooted in the Social Ecological Model (SEM) of behaviour change, investigated the structural and demand-side cognitive barriers that limit antenatal and family planning care-seeking behaviour among households in Gombe State, Nigeria.

**Method:**

Qualitative in-depth interviews, each lasting 60–70 min, were conducted with 56 respondents, including mothers/expectant mothers (n = 15), their household decision-makers/spouses (n = 16), community leaders (n = 16), and primary healthcare centre (PHC) service providers and volunteers (n = 9). The qualitative instrument was developed and analysed based on four core elements of the SEM: intrapersonal, interpersonal, institutional, and community-level factors of behaviour. The study was conducted in Dukku, a rural local government area (LGA), and Yamaltu Deba, a peri-urban LGA.

**Results:**

The findings indicate that the direct and indirect costs of care, service quality, and PHC proximity were the main supply-side and institutional-level factors. Most of the demand-side barriers were associated with individual (poor salience), relational (limited spousal support), and community-level (traditional maternal practices) factors of the SEM model. Optimism bias, overconfidence bias, and present bias are some demand-side psychological barriers that impede maternal care uptake. Similarly, supply-side barriers, such as poor quality of care, can significantly undermine individual and community-level demand for PHC-based maternal care.

**Conclusion:**

The SEM model offers a comprehensive framework for understanding the complex, multi-layered factors influencing care-seeking behaviours toward antenatal care and family planning. The findings show that although relational and community-level barriers are prominent barriers to care-seeking, they are all interlinked and are fuelled by a broad scope of institutional-level barriers.

## Background

Maternal morbidity and mortality remain critical global issues, with an estimated 287,000 women dying during and after pregnancy and childbirth in 2020 [1]. While maternal mortality has declined in high-income countries, low- and middle-income countries (LMICs) accounted for over 90% of maternal deaths in 2020 [2]. Sub-Saharan Africa (SSA) has some of the highest maternal mortality rates [3, 4], with Nigeria reporting 1,047 deaths per 100,000 live births in 2020 [4]. These high rates of maternal mortality and morbidity negatively affect the welfare of newborn children but also have severe socioeconomic consequences for households and communities, such as loss of income for the household and diminished productive labour in key sectors like agriculture that benefit from female labour [5–7].

Access to skilled birth services and maternal care, including antenatal check-ups, counselling, and screening at primary healthcare facilities, has been shown to significantly reduce maternal and child morbidity and mortality in LMICs [6–8]. However, limited access to and utilisation of maternal care services at primary healthcare centres in LMICs is driven by both demand- and supply-side factors. Supply-side challenges in Southeast Asian LMICs, including Myanmar, the Philippines, and Pakistan, involve poor service quality, inadequate medical resources, non-compliance with health service delivery guidelines, weak data systems, geographic barriers, and high transport costs [9–13]. African countries such as Nigeria, Ghana, and Kenya face similar issues, compounded by severe healthcare worker shortages and poor attitudes toward service delivery [14–18]. Prominent barriers in African countries include religious norms promoting home births, limited female autonomy in health decisions, and low awareness of maternal care services [12–14, 18, 19]. At a high level, most demand-side barriers across LMICs in Africa and Asia revolve around low household income, low education levels, mistrust of modern healthcare, poor symptom recognition, and cultural norms favouring home-based maternal care [9, 11–19].

Despite extensive research on barriers to maternal care utilisation, few studies have explored the psychological factors underlying these barriers, especially in SSA. Most research on the psychological aspects of maternal health focuses on women in high-income countries, leaving a gap in behavioural studies in SSA. Recent formative research highlights the interplay between psychology and health economics, showing how cognitive biases affect maternal care demand [20, 21]. For instance, optimism and pessimism influence delivery options, with dispositional optimism linked to worse health outcomes and pessimism having a protective effect on health-seeking behaviour [22–24]. Present bias, where individuals prioritise immediate gains over future health, has been associated with delayed health-seeking, especially among expectant mothers who delay routine maternal checks until complications arise [25, 26]. Additionally, negative past experiences with institutional maternal care deter future use of these services [27].

Given the spectrum of factors that influence maternal health-seeking behaviour, this study utilises the Social Ecological Model (SEM) developed by Bronfenbrenner [28] to unpack and understand some factors hindering health-seeking behaviour. The application of this model seeks to enhance the understanding of the barriers to maternal health-seeking behaviour by examining the interaction between structural supply-side and psychological demand-side factors affecting antenatal care (ANC) and family planning (FP) uptake at primary health centres (PHCs) in Gombe, Nigeria. The SEM model identifies five core levels influencing decision-making: intrapersonal (individual) level, interpersonal (relational) level, institutional (organisational) level, community level, and policy level [28–30]. This study focuses on the first four levels—individual, relational, institutional, and community. The individual level of the SEM represents individuals’ characteristics, including knowledge, attitudes, beliefs and practices that weigh on decision-making. The interpersonal or relational level of the SEM describes how individuals’ familial and social relationships impact health-care decision-making. The institutional level describes the roles and influence of service providers and health facilities on health-seeking behaviour. Finally, community-level factors can be loosely described as the effect of social influences on decision-making, such as community leaders, widely held community beliefs, practices, and traditions [29–31].

This study categorises SEM-level barriers into supply- and demand-side factors, with a focus on the behavioural and cognitive biases that influence certain demand-side barriers. The study aims to answer the following research questions: (a) What is the level of knowledge about maternal care services, including antenatal care and family planning, among women and their household decision-makers? (b) What are women’s experiences seeking maternal care at PHCs? (c) What cultural and religious beliefs affect maternal care and FP? (d) What are the experiences of primary health care providers in delivering maternal care?

## Methods

### Study design

This qualitative research approach consisted of In-Depth Interviews (IDIs) to explore key supply- and demand-side barriers to accessing maternal care services, guided by the four core elements of SEM (individual, relational, community, and institutional levels barriers). The study team followed the Consolidated Criteria for Reporting Qualitative Research Guidelines (COREQ) in the design and implementation [32]. The study focused on gathering qualitative insights from Gombe, northeast Nigeria.

### Study sample

The participants included recipients of maternal care (mothers and expectant mothers), household decision-makers who influence health-seeking behaviour (husbands and mothers-in-law), community influencers (traditional and religious leaders), and maternal service providers such as Community Health Influencers, Promoters, and Service agents (CHIPS), along with PHC maternal staff (nurses). A total of 56 participants took part in the qualitative study, with the sample size determined by the anticipated saturation level for each respondent category [33].

Purposive sampling was used to recruit respondents who met the following criteria: pregnant women aged 18 or older, mothers aged 18 or older with young children (0–5 years), spouses of the recruited expectant mothers or mothers, community leaders (such as traditional village heads and religious leaders), and personnel providing maternal care services or support at a PHC, including community health volunteers. All recruited respondents provided both verbal and written consent. Community health volunteers and PHC maternal care staff were identified for the study by Gombe State Primary Health Development Agency (GSPHCDA). PHC staff were recruited in the study locations based on their years of experience and availability. Community leaders, mothers, expectant mothers and their respective spouses were identified via community health volunteers and recruited using door-to-door canvassing in the study locations.

### Data collection and questionnaires

Four semi-structured qualitative questionnaires/guides were created for the four respondent categories outlined in the Study Sample section. These guides, designed to explore barriers to maternal health-seeking behaviour using the SEM framework, were initially developed in English and translated into Hausa to ensure comprehension. The interview guides were piloted with two respondents from each category, and feedback from these pilots was used to refine the guides. However, data from the pilot interviews were not included in the final study findings.

The interviews were conducted by trained and experienced qualitative researchers and public health professionals. The respondents were offered the choice to be interviewed at a central public location, at a PHC or at their homes. Each respondent received an overview of the study objectives and had the opportunity to ask questions before, during and after participation. Interviews lasted between 60 and 70 min and were recorded, transcribed, and translated into English. Study team members reviewed the transcribed data before synthesis. Data collection took place from September to October 2022.

### Study location

The study was conducted in two local government areas: Dukku (rural) and Yamaltu Deba (peri-urban) in Gombe State, Nigeria. Gombe state was chosen as the primary study location because it is located in Northeast Nigeria, a region with relatively high maternal and child mortality (1047 deaths per 100,000 live births in 2020 [4]) but has comparatively lower levels of insecurity compared to other states in the region [34].

### Data analysis

The qualitative data from the IDIs were analysed using thematic analysis, which consisted of analysing and reporting patterns within data and grouping them under relevant recurring themes [35]. Each interview transcript was reviewed and validated by a second researcher in the study team. The transcripts were coded manually using a data stripping matrix in Excel and also with NVivo to collate and identify concepts and insights around barriers to maternal care uptake that address the research questions. Codes were developed inductively based on the data gathered, and all qualitative themes were formed inductively. Thematic analysis was used to categorise the qualitative insights under the 4 SEM levels of interest (individual, relational, institutional, and community) and distilled into supply-side or demand-side thematic groups (Table 1). Behavioural science literature was used to draw parallels between demand-side themes and common psychological/cognitive biases. In this study, we link behavioural biases to qualitative insights that align with common biases identified in the literature on health-seeking behaviour. In other studies, these biases have been identified by observing actions and communication that reflect behavioural traits in the field or controlled experiments [36]. For instance, optimism bias is observed through expressed beliefs and actions that anticipate positive outcomes while overlooking risks [37].

**Table 1:** Thematic breakdown of the qualitative guides and response categories

Qualitative themes explored		Relevant respondents			
SEM categories	Description	Service providers	Household decision-makers	Mothers and expectant mothers	Community influencers
Individual	Knowledge, perceptions, beliefs, practices and pain points toward ANC and FP maternal services uptake		✔	✔	
Relational	Household awareness and influence on maternal health seeking		✔	✔	✔
Institutional	Institutional challenges in the provision of quality care and perceived quality of maternal care provision	✔		✔	
Community	Traditional and cultural norms that influence maternal health-seeking		✔	✔	✔

## Results

### Respondent description

The study involved 56 respondents across four categories: 16 household decision-makers, 16 community influencers, 15 mothers or expectant mothers, and 9 PHC service providers (Table 2).

**Table 2:** Sample breakdown of respondents in Gombe, Nigeria (n = 56)

Participant category	Description	N (%)
PHC service providers	Personnel who delivered or supported service delivery for maternal services at PHCs	9 (16.0%)
Household decision makers	Husbands, mothers-in-law, and fathers-in-law that have a significant influence over the health decision-making of mothers and expectant mothers in their homes	16 (28.6%)
Mothers and expectant mothers	Women with adolescent children and pregnant women	15 (26.8%)
Community influencers	Traditional leaders who understand the cultural and social dynamics of their community and influence healthcare-seeking behaviour	16 (28.6%)

Most mothers and expectant mothers interviewed were from Yamaltu Deba LGA (n = 8, 53%). The women interviewed were predominantly aged 26–30 years (n = 6, 40%), with most having secondary education (n = 5, 33%) as their highest level of education. Most mothers and expectant mothers were either unemployed (n = 6, 40%) or engaged in petty trading (n = 6, 40%). All participants were married with at least one child, except for one widow. Most of the respondents had 0–2 children (n = 7, 47%), all figures are displayed in Table 3.

**Table 3:** Demographic breakdown of mothers and expectant mothers

Expectant mothers and mothers (n = 15)				
Characteristics		Local government area (LGA)		Total
		Dukku (n = 7)	Yamaltu Deba (n = 8)	
Type	Expectant mother	4	4	8
	Mother	3	4	7
Highest education	No education	1	0	1
	Primary education	2	0	2
	Secondary education	1	4	5
	Islamic/Quranic education only	3	1	4
	Tertiary education	0	2	2
Occupation	Trader	1	5	6
	Business owner	1	1	2
	Employed	0	1	1
	No occupation	5	1	6
Age	20–25	1	1	2
	26–30	4	2	6
	31–35	2	3	5
	36–45	0	2	2
Marital status	Married	7	7	14
	Widowed	0	1	1
Number of children	0–2	2	5	7
	3–5	3	0	3
	6 or more	2	3	5

Household decision-makers were predominantly husbands (n = 10, 63%), with primary education being the highest level of schooling for most. Most husbands interviewed were petty traders. The average age range for husbands was 38–40 years, while mothers-in-law were generally between 50 and 56 years old (Table 4).

**Table 4:** Demographic breakdown of household decision-makers

Household decision makers (n = 16)				
Characteristics		Local government area (LGA)		Total
		Dukku (n = 6)	Yamaltu Deba (n = 10)	
Type	Husband	4	6	10
	Mother	2	4	6
Highest education	No education	2	1	3
	Primary education	3	5	8
	Secondary education	1	1	2
	Islamic/Quranic education only	0	2	2
	Tertiary education	0	1	1
Occupation	Trader	3	5	8
	Business owner	1	1	2
	No occupation	2	4	6
Age	Average age of husband (in years)	38	40	39 (average)
	Average age of mother-in-law (in years)	50	56	54 (average)

Community influencers included local religious leaders (6, 38%), traditional leaders or village heads (5, 31%), and community volunteers (5, 31%). Each community leader had completed either primary (4, 25%), secondary (4, 25%), or tertiary education (4, 25%) or the equivalent of Islamic/Quranic education (4, 25%). The average age of these community influencers was 54 (Table 5).

**Table 5:** Demographic breakdown of community influencers

Community influencers (n = 16)				
Characteristics		Local government area (LGA)		Total
		Dukku (n = 5)	Yamaltu Deba (n = 11)	
Type	Community leader/village head	2	3	5
	Religious leader	2	4	6
	Community Volunteer	1	4	5
Highest education	No education	0	0	0
	Primary education	2	2	4
	Secondary education	1	3	4
	Islamic/Quranic education only	2	2	4
	Tertiary education	0	4	4
Age	Average age	54 years	54 years	54 years

The service providers interviewed included 5 PHC staff members (nurses, midwives, and clinicians) and four community volunteers, known as Community Health Influencers and Promoters Service agents (CHIPS), who assist the community in accessing basic care at local PHCs. All CHIPS agents had secondary-level education, while PHC staff had tertiary-level education. The CHIPS agents had at least two years of service experience, and PHC staff had at least five years of experience in primary healthcare service delivery (Table 6).

**Table 6:** Demographic breakdown of service providers

Service providers (n = 9)				
Characteristics		Local government area (LGA)		Total
		Dukku (n = 5)	Yamaltu Deba (n = 4)	
Type	CHIPS agents	2	2	4
	PHC service providers	3	2	5
Highest education	Secondary	3	2	5
	Tertiary	2	1	3
	Islamic education only	0	1	1
Service duration	0–5 years	2	3	5
	6–10 years	2	0	2
	11 years or more	1	1	2

### Demand side-barriers

#### Awareness of PHC maternal care services

Most mothers were familiar with the concept of a PHC and the spectrum of services available at their local PHCs, including ANC and FP. Those who knew what a PHC was drew comparisons to “a small hospital” or identified it as a place to access ANC and child delivery services, immunisation, and treatment for malaria. One mother reports:

*“Primary healthcare centres are where you get first healthcare before you go to bigger hospitals.” (Mother, 25, Yamaltu/Deba, Gombe State)*

Additionally, many women and household decision-makers understood what FP is and believed it was important for child spacing and reducing the risk of unwanted pregnancies, especially after birth.

*“You will see a woman getting pregnant when her child is barely a year old and even lose the pregnancy at the end, so for these reasons, it [FP] is good and important.” (Mother, 45, Yamaltu/Deba, Gombe).*

Respondents showed greater awareness of ANC than FP. Women reported that they needed more information on FP services, particularly around long-lasting contraceptives, and how long injectable contraceptives last.

*“No, I don't have enough information about family planning, but I want to know more. I want to know all because I would like to use one.” (Expectant Mother, 27, Dukku, Gombe)*

*“It (FP) is not important because God is the one who gives children and when to deliver them.” (Expectant Mother, 23, Dukku, Gombe)*

*“I don’t have enough information about FP because I don’t understand it.” (Expectant Mother, 23, Dukku, Gombe)*

Overall, awareness and understanding of PHC maternal services was less prominent among women residing in Dukku (a rural LGA) than those in the peri-urban LGA, Yamaltu Deba.

Household decision-makers, particularly spouses, reported low levels of awareness regarding PHC maternal care services. Many were unfamiliar with the services offered and could not describe what antenatal care entails. Unlike the trends observed among mothers and expectant mothers, awareness among decision-makers (predominantly husbands) was lower in the rural LGA compared to the peri-urban study location, Yamaltu Deba. One husband in Yamaltu Deba reports.

*“I am not sure of ANC services. I just know a PHC is like a small hospital for treatment of malaria, fever and blood tests.” (Husband, 33, Yamaltu/Deba, Gombe)*

#### Attitudes and beliefs toward PHC maternal care services

Despite a relatively good awareness of ANC and FP services among mothers, their attitudes toward utilisation were largely complacent, with minimal urgency for regular ANC. Their approach to maternal care tended to be more curative than preventive, resulting in infrequent ANC visits and health-seeking behaviour at PHCs being limited to emergencies, such as severe malaria. Few women reported giving birth in health facilities or consistently attending the recommended eight ANC appointments during pregnancy. FP was regarded as an even lower priority compared to ANC and was not actively pursued. Health-seeking at PHCs was often delayed until alternative care methods, such as traditional remedies or self-medication through local or proprietary medicine vendors, proved ineffective.

*“Formal family planning is not a common thing in this area.” (Traditional leader, 50, Dukku)*

*“All the women in my family gave birth at home, and nothing happened to them, no ANC.” (Husband, 41, Dukku)*

Some women reported downplaying the probability of experiencing maternal complications. This sense of optimism leads individuals to a biased belief that they are more likely to experience positive outcomes than negative ones [38]. This perception diminishes the perceived need for ANC and FP services at PHCs.

*“If I feel there is any complication, then I will go (for ANC), but thank God I do not think I need to.” (Mother, 30, Yamaltu Deba)*

The comment above shows a preference for curative over preventative healthcare services, which may indicate a tendency to invest minimal effort in preventive care in the short term. This trade-off between present and future health-seeking behaviour could be explained by present bias, where individuals prioritise immediate benefits over future ones [39].

Additionally, community-held beliefs, often based on misinformation, influenced individual attitudes toward maternal health-seeking, particularly around FP. A prevalent misconception was that FP implants inserted into the vagina could disappear and destroy the womb.

*"The method of FP is good, but I am scared because of the implant. Some said if inserted it won't come out, and it causes bleeding. " (Expectant Mother, 27, Dukku, Gombe)*

*“There was this belief from our parents before we got married. They said anytime women went for ANC and were given drugs, the unborn child would grow so big in the womb of his mother, which must lead to an operation during delivery.”(Husband, 40, Dukku, Gombe).*

Some respondents echoed common religious narratives that contraceptive methods were against the will of God because they restrict the blessing of children.

*“[FP] is not important because God is the one who gives children and when to deliver them.” Expectant Mother, 23, Dukku, Gombe)*

The comment above shows the need for myth-busting campaigns to address misconceptions associated with ANC and FP usage. Campaigns led by influential community leaders are more likely to be credible [40] and show FP as a safe method to space children and ensure children are born when the parents are ready to receive them rather than a means to restrict the choice to conceive.

#### Household decision-making around maternal care

Awareness, attitudes, and beliefs about maternal care are critical, but especially important for household decision-makers. Most women indicated that their husbands were the primary and sole decision-makers regarding where the household sought medical care. Joint decision-making between women and their spouses was also commonplace, however, most women deferred to their spouses to make the final decision around health seeking.

*“My husband does all the decision-making for medical decisions. My husband does all that since I am under him in the house.” ( Expectant mother, 27, Yamaltu Deba, Gombe)*

*I have a family and a wife. When my children are sick, or if my wife is sick, I decide which hospital to take them to. My wife can also decide, when it comes to the issue of which of the healthcare centres we need.” (Husband, 31, Dukku, Gombe).*

Husbands were not only the primary healthcare decision-makers, but many women also reported relying on them, along with other family members like mothers and siblings, for guidance on everyday life issues.

*“I go to my husband (for advice), he guides me in the right direction.” (Mother, 29 Dukku, Gombe)*

*“I go to my elder sister (for advice) because she got married before me and knows more about life than me.” (Expectant Mother, 27, Dukku, Gombe)*

Although the husbands (primary household decision-makers) from the study sample were not as knowledgeable about ANC and FP as their spouses, they understood the importance of professional medical care. They considered many factors before permitting their households to seek care at a specific health facility. These factors included perceived competence of staff, distance, and cost, with a few spouses admitting to sending their households to secondary or specialist hospitals if the local primary health centres were not perceived to be well-staffed or equipped.

*“The thing I consider first is the performance and the competence of the institution. I check how well their staff and health workers treat patients. I don’t necessarily bother about the distance or the bills first.” (Husband, 33, Yamaltu Deba, Gombe).*

*“The main thing I considered when choosing health care was the closeness to my home. Healthcare should be easy to access in no time.” (Husband, 31, Dukku, Gombe)*

Household uptake of medical care was also based on perceived need or severity. For example, getting a sick child to a health facility would be deemed more important than routine ANC visits. This suggests the need to tailor maternal care interventions to address the knowledge gaps among men and emphasise the criticality of pre-emptive and preventive routine maternal care, such as ANC.

#### Alternatives to PHC-based maternal care

The low health-seeking behaviours among mothers and expectant mothers were strongly influenced by existing community traditions related to childbirth and a preference for traditional alternatives to PHC-based maternal care. Home deliveries with traditional birth attendants (TBAs) and use of herbal remedies in place of PHC-prescribed maternal drugs and supplements were a common practice. Nearly half of the women interviewed reported using traditional herbal methods for FP, such as herbal tinctures like pumpkin seeds, as shown below:

*“Traditional remedies are taken after sex, then it goes in to melt the sperm so it doesn’t stay active, then another one is mixed with salt and kashin zomo [rabbit faeces].” (Expectant Mother, 27, Yamaltu/Deba, Gombe).*

The use of TBA and traditional remedies was occasionally coupled with religious traditional practices, which represented another layer of the community-level barriers to formal maternal care at a PHC. Some of these practices included herbal remedies, specifically from religious medicine vendors for safe delivery or relying on spiritual items such as beads for the prevention of unwanted pregnancies and consuming liquids in which passages of the Quran are soaked to aid a healthy pregnancy. One respondent reported:

*“In my place, there is charm, or something tied on the waist which they remove whenever women want to get pregnant.” (Expectant Mother, 23, Dukku, Gombe)*

*“Arabic [from the Quran] written on slates, washed and given to women to drink has been used for a healthy birth.” (Expectant Mother, 35, Dukku, Gombe)*

*“[For FP] there are local seeds of trees that they [traditional medicine vendors] mix and they also provide other Islamic medicine for women.” (Expectant Mother, 32, Yamaltu/Deba, Gombe)*

Some traditional leaders acknowledged that traditional healthcare practices did hinder maternal health-seeking at a PHC. However, religion was not perceived as a significant barrier for most community members who were literate and well-informed on the importance of PHC-based care, as the comments from community leaders show below:

*“For culture and traditional practices, I’ll say yes. It hinders people from accessing the hospital as it promotes more traditional medicine but religion doesn’t really hinder anyone from accessing the hospital.” (Community leader, 55, Dukku).*

*“Sometimes, people don’t believe in healthcare because of religion and illiteracy. But, with awareness, we do convince religious people to seek care.” (Community leader, 62, Yamaltu Deba)*

Reliance on alternatives to PHC maternal care services can distort the perceived value of primary healthcare at a PHC. It is important to consider how to counter such habits through evidence-driven initiatives that showcase the value of ANC and FP over unvalidated traditional alternative methods of maternal care. The next section discusses the findings on the supply-side barriers.

### Supply-side barriers

Most of the supply-side barriers were present at the institutional level. These institutional-level barriers highlight the experiences of women in accessing PHC maternal services, coupled with PHC service provider challenges in delivering quality maternal care. Below are some of the barriers that the respondents identified.

#### Cost of maternal care and PHC proximity

The cost of PHC services was the most frequently cited barrier to regular ANC uptake. This cost burden included direct expenses (such as service fees and medication) and indirect costs (like transportation to and from the health facility).

*“It is expensive, the amount is five hundred naira (per ANC visit) and some people don't go because of the price (of transport). They should reduce it to two hundred Naira.” (Expectant Mother, 30, Dukku, Gombe).*

Transportation costs were a major issue for Dukku residents due to its larger geographical spread and fewer functional PHCs (17) compared to Yamaltu Deba. The average distance to the nearest PHC in Dukku was much greater than in Yamaltu Deba, with the farthest being 40–60 min away by commercial motorcycle. ANC visit fees ranged from NGN1000 to NGN2000, which is relatively affordable on a national scale, however, some respondents reported these fees varied considerably, most likely due to inflationary pressures and noted additional ‘under-the-table’ payments to avoid service delays. Rising transportation costs coupled with unpredictable costs of care discouraged consistent health-seeking, particularly among those with lower household incomes.

#### Quality of care and availability of medical resources

A major institutional barrier to seeking ANC and FP services at PHCs was the poor quality of care, primarily due to limited resources and staff shortages. Despite Dukku’s population being over 207,000 at the last 2006 census [41], there were only 17 functional PHCs, each potentially serving over 5000 people during the implementation of this study. This strain on resources led to long wait times for ANC appointments and complaints from women about insufficient staff. Additionally, shortages of beds, essential ANC commodities like folic acid and iron supplements, and poorly maintained equipment, such as sonographic scanners, further compromised care quality. Below is a comment from an expectant mother echoing the sentiment of some respondents:

*“My experience was not encouraging because they kept us waiting for a long time unattended, and I was very hungry by then. I promised not to go back to the hospital because they did not treat us well and did not give us anything.” (Expectant Mother, 23, Dukku, Gombe).*

In Dukku LGA, concerns about healthcare worker absenteeism and limited accessibility to commodities (Iron and folic acid supplementation for ANC visits, FP pills, etc.) needed for the provision of maternal services were more pronounced. Absenteeism among maternal care providers in these areas exacerbated the pressure on already overburdened staff. The comment below by an expectant mother shows how these provider dynamics might affect service delivery for those in need:

*“Sometimes they don’t attend to people in time and also refer to some patients as dirty people. You sometimes feel like never visiting the PHC again.” (Expectant Mother, 35, Dukku, Gombe)*

The comments above show the frustrations some mothers have when accessing primary health care. These negative patient experiences discourage many mothers from seeking care and interacting with providers who are deemed as unapproachable or unfriendly. Service provider attitudes toward patients was an acute pain point for many of the mothers interviewed. One expectant mother expressed:

*“Stop shouting at patients because no one visits the PHC for fun but for medication. If not for persistent pains or ailments, some women wouldn’t have gone back to the PHC again.” (Expectant Mother, 35, Dukku, Gombe).*

#### Capacity of service providers

Despite the relatively extensive professional and technical experience of the healthcare providers interviewed, many noted they lacked adequate training on soft skills like bedside manner, interpersonal skills, and patient empathy, leaving them unprepared to manage challenging patients or working environments:

*“We could benefit from better pay, training on how to handle high-pressure scenarios, and how to deal with difficult patients. How to manage patients basically.” (Service provider, Dukku)*

The lack of interpersonal skills among overworked and often underpaid staff resulted in occasionally harsh and unempathetic care. This lack of empathy discouraged some women from returning to PHCs and adhering to recommended ANC visit schedules. This highlights the need for regularised pay for PHC staff and tailored interventions to enhance providers' soft skills, such as emotional competence, to improve provider–client interactions. The next section discusses the implications of these findings, along with the study’s limitations and areas for future research.

## Discussion

The synthesis of the qualitative findings with the aid of SEM revealed supply-side and demand-side barriers to utilising ANC and FP at PHCs. The supply-side constraints are largely driven by institutional-level factors that limit the quality of care available. On the other hand, demand-side barriers are characterised mainly by individual, relational, and community-level factors, which consist of awareness, beliefs, perceptions, community norms, and practices that shape decision-making in accessing maternal care. These findings indicate areas that can be addressed to increase the demand and uptake of maternal care services, as further discussed below.

### Demand-side barriers

Lack of salience around PHC-based maternal care services among women and their spouses is among the leading individual-level and relational-level barriers to maternal service utilisation [2, 4, 9, 13, 17]. Despite the familiarity of most women with ANC and FP services, self-reported ANC attendance was inconsistent at best. This suggests that knowledge or awareness that a service exists does not mean it will be utilised [6]. Salience is the missing link in optimising awareness campaigns for desired behaviour change [42–44]. Salience in our context can be defined as the perceived importance placed on maternal care knowledge. This study has shown a myriad of factors contribute to poor salience such as conflicting traditional beliefs around maternal care, misinformation about the side effects of institutional care, alternative health practices, past negative experiences with medical providers, and limited spousal support all contribute to poor salience [12, 45–47]. Both women and their spouses could benefit from targeted advocacy that not only provides knowledge about PHC-based maternal care but also creates more emphasis on the need for spousal support in championing maternal care uptake and the dangers of maternal care neglect [44, 46] or potential losses to households that do not prioritize maternal care.

The study also revealed that the complacent attitude toward low ANC uptake was mostly among women who had no history of pregnancy complications or had few interactions with women who had suffered complications. This suggests some of the women in our sample may have had a low perceived risk of pregnancy complications due to past experiences. A similar study in Kaduna, Nigeria, found that women without previous obstetric complications tended to overlook maternal health services [17], which could be a form of optimism bias, a cognitive error where individuals underestimate their risk of experiencing adverse outcomes [48]. Optimism bias has been linked to a woman’s likelihood of undergoing unplanned caesarean section delivery, COVID-19 care, sexual health screening and perceived cardiovascular health [22, 49–51]. It is also plausible to assert that inaccurate symptom recognition could also be playing a role in low perceived risks women associate with their gestational period. Symptoms such as headaches, bleeding and fatigue that require monitoring by a medical professional may be normalised and only investigated when symptoms become too acute [12, 17].

Alongside individual-level biases, community-level practices and beliefs also had a significant influence over health-seeking behaviour, particularly community-wide preference for alternative maternal care, such as traditional birth attendants and herbal or religious remedies [15, 18, 52]. Although traditional birth attendants have supported rural communities across West Africa for many generations and have gained credibility, few TBAs are formally trained and many rely on personal experience rather than medical training [53]. Self-proclaimed TBAs are often trusted without question [15, 18, 52, 53], and this unwavering trust often fosters a biased overconfidence in the abilities of TBAs to manage all gestational concerns. This Overconfidence bias [54] is often studied regarding service provider errors in diagnostics and quality of care [55], however, some research indicates it could be a plausible driver of poor health-seeking behaviour [20, 25, 54, 56, 57].

A common theme across all individual, relational, and community-level demand-side barriers is the preference for curative rather than preventative health-seeking, consistent with literature [15, 18, 52]. While most households in our sample are aware of PHC-based maternal services, utilisation tends to occur only in critical situations rather than as a regular practice throughout the gestational period. Factors such as low household income, unaffordable maternal care costs, geographic constraints, and easily accessible traditional alternatives contribute to this trend. Psychologically, the trade-off between preventative and curative health-seeking can be explained by present bias, where immediate short-term gains (time and cost savings) of low maternal care uptake are prioritised over future/long term benefits of consistent maternal care uptake [58]. Studies indicate that the propensity to make beneficial health choices depends on the preference for future versus present gains [25, 26, 59]. In this study's context, present-biased households may view the current costs of ANC and FP as higher than the future costs of unplanned pregnancies or birth complications [58, 60]. This bias negatively affects how households prioritise health-seeking, impacting both short-term and long-term maternal and child health outcomes.

### Supply-side barriers

The cost of maternal services at PHCs is a leading reason for maternal care neglect among women in the study sample. Similar studies indicate that costs are a significant barrier to initial maternal care uptake, particularly among rural households in SSA [45, 46]. Proximity and transportation costs also pose considerable challenges, especially during off-peak times when public transportation is unavailable in remote areas [47]. Costs may hinder initial engagement with PHC-based maternal care services, but poor PHC service quality and delivery discourage continued or repeat usage of maternal services [45, 47]. The concerns with maternal care quality hinged on 3 factors: service attitude, service delivery promptness and PHC resources. The lack of PHC resources, such as tools for sonography, maternal supplements, beds, etc., can be remedied easily through more state-level funding. However, improving service delivery attitudes requires both health system investments and behaviour change interventions [61].

Poor service delivery at PHCs contributes to negative perceptions about the care quality, which are then communicated through interpersonal and community networks [39, 46, 62]. Poor service delivery attitudes are often due to inadequate training on soft skills around patient care as well as staff shortages, which strain existing human resources, making it challenging to provide patient and attentive care [18, 61, 63, 64]. Assessments of healthcare service quality in Nigeria [65] and South Africa [66] also suggest that the absence of disciplinary actions in response to poor service delivery creates a culture of negligent patient care and poor supportive supervision. Interventions that encourage and reward service delivery performance have been found to incentivise better quality care [67]. Thus, the supply-side barriers could be addressed through capacity-building initiatives, such as training on interpersonal skills, performance-based incentives, and government interventions to reduce the ratio of providers to the public.

The interlinkages of barriers across the SEM model signify the need for behaviour change interventions at all levels of the SEM. Individual and relational level interventions could involve closing the disparity in spousal understanding of maternal care and making consistent care more salient. At the institutional level, reforms to improve the quality of care could involve developing quality care guidelines, implementing supportive supervision for service providers, and building referral systems that allow overburdened facilities to give patients more options for attentive care at other neighbouring facilities [9, 10, 12, 68, 69].

### Study limitations

The main limitation of this study is that purposive sampling was used to select respondents, and thus their experiences and opinions may not represent most households in Gombe State. Additionally, the study captured only a limited range of maternal care service providers, primarily health volunteers and nurses. This limited variation in interviewed service providers may have reduced the nuance in the institutional-level barriers identified.

## Conclusion

This study offers a unique perspective on barriers to maternal care by mapping SEM-based demand- and supply-side challenges and highlighting the behavioural and cognitive biases that underlie these issues. Using the SEM enables a holistic approach to synthesising qualitative data and unpacking the factors across all levels of society that influence the decision to seek maternal care at a PHC. The findings show that although individual, relational and community-level barriers are prominent, they are all interlinked with each other and are fueled by existing broad-scoped institutional-level barriers such as cost and quality of care which have far-reaching impacts on demand-side health-seeking behaviours.

### Areas for further research

This study examines the supply- and demand-side barriers to the uptake of ANC and FP services. While addressing these barriers and fostering new health-seeking habits is possible, it can be challenging, especially if preferences are deeply ingrained [60]. Further research could explore interventions to tackle these barriers. Additionally, another area for investigation could involve co-creating solutions with community members and key stakeholders in Gombe State to effectively address these challenges.

## Data Availability

The datasets generated and analysed during this study are not publicly available due to the privacy clause stipulated in participant consent. The qualitative tools used are available upon request from the corresponding author.
